# Supplementary material for: Mosquito viromes across England and Wales reveal hidden arbovirus signals and limited ecological structuring
Source: Front Microbiol. 2026 Jan 22;17:1749228. doi: 10.3389/fmicb.2026.1749228 (PMC12872884; doi:10.3389/fmicb.2026.1749228)
Supplement: Supplementary file 1 [file Data_Sheet_1.PDF]

<u>Supplementary Table S1. Summary of Culex sampling sites and pool characteristics</u>

Site number	Land Type	Latitude	Longitude	Total mosquitoes processed	IDs
104	Urban	51.6156132	-3.94996639	6	104.1<br>104.3<br>104.4<br>104.5<br>104.6<br>104.7
105	Rural	53.3244639	-4.426866689	3	105.1-105.3
113	Rural	50.54636699	-4.31608799	1	113.3
114	Urban	50.38598919	-4.11885559	1	114.1
118	Urban	54.5403933	-3.562480588	1	118.1
119	Rural	53.8855015	-2.736061188	2	119.1, 119.2
120	Urban	54.0411293	-2.791501188	1	120.1
122	Rural	53.404711	-2.933357588	2	122.2, 122.3
123	Rural	53.2252941	-3.480024789	2	123.1 123.2
130	Urban	51.4816671	-3.188956689	7	130.1 130.3 130.5
136	Urban	53.2540362	-2.514392588	1	136.1
138	Urban	52.7025155	-2.508510888	3	132.1 132.2 132.8
139	Rural	52.2332384	-2.580829788	1	139.2
142	Urban	51.5894787	-2.975680489	7	142.1<br>142.3<br>142.4<br>142.5<br>142.6<br>142.8<br>142.9
144	Urban	50.62687589	-2.472464789	3	144.1-144.3
149	Rural	53.9716919	-2.175998787	6	149.6
150	Urban	53.7943875	-1.259113287	4	150.1<br>150.3<br>150.4<br>150.6
154	Urban	53.0539454	-2.195419888	6	154.4<br>154.7<br>154.20<br>154.21<br>154.22<br>154.24
157	Rural	52.2949804	-2.209401088	1	157.1
162	Urban	51.087065	-1.794669989	4	162.1 162.2 162.4
164	Urban	50.72535339	-1.855965789	1	164.2
167	Rural	54.0721079	-1.284428887	2	167.1 167.2
168	Urban	53.8002586	-1.518813087	4	168.1-168.4
173	Rural	52.6787999	-1.053540987	1	173.5
179	Rural	51.2503628	-1.512127988	1	179.1
180	Urban	51.3938272	-1.319087488	7	180.1-180.7
181	Rural	50.85711469	-1.591529689	1	181.3
182	Urban	50.93676339	-1.376515088	6	182.1<br>182.2<br>182.4<br>182.5<br>182.6
188	Urban	53.2088378	-0.557121787	4	188.1<br>188.3<br>188.5<br>188.6
190	Urban	52.5834301	-0.237322387	5	190.2 190.4 190.5
191	Rural	52.2773174	-0.487993287	5	191.1-191.3

194	Urban	51.5421868	-0.243651188	9	194.1<br>194.2<br>194.3<br>194.4<br>194.6<br>194.7<br>194.8<br>194.9<br>194.10
195	Suburban	51.3877691	-0.233374188	4	195.3<br>195.4<br>195.6<br>195.7
196	Urban	51.4950369	-0.270840988	4	196.1-196.4
197	Rural	50.75413649	-0.765635088	6	197.1<br><br>197.2<br>197.4<br>197.7<br>197.8 197.13
198	Urban	50.84029749	-0.761142188	8	198.1<br>198.2<br>198.3<br>198.4<br>198.5<br>198.6<br>198.7<br>198.9
206	Urban	51.5729761	-0.096069787	6	206.1<br>206.2<br>206.3<br>206.6<br>206.7<br>206.8
211	Rural	50.92130619	0.336089712	8	211.1<br>211.2<br>211.3<br>211.5<br>211.6<br>211.7<br>211.8<br>211.9
212	Urban	52.6330027	1.309213814	8	212.1<br>212.2<br>212.3<br>212.4<br>212.5<br>212.7<br>212.8<br>212.9
218	Urban	51.3788279	1.394657113	1	218.1
301	Rural	52.9021736	0.006476413	1	301.1
303	Rural	54.0841747	-3.237748188	1	303.1
305	Rural	53.8884569	-3.029963788	8	305.2<br>305.3<br>305.4<br>305.5<br>305.6<br>305.7<br>305.8<br>305.10

306	Rural	51.8537936	1.223001713	4	306.1<br>306.2<br>306.3<br>306.5
307	Rural	50.53773849	-4.934777691	1	307.2
308	Rural	52.8876117	-4.107689989	2	308.1 308.3
310	Rural	54.7505281	-3.447008788	3	310.3<br>310.4<br>310.13
314	Rural	55.6742646	-1.800676886	4	314.1<br>314.3<br>314.4<br>314.7
317	Rural	51.4576967	-0.547095088	7	317.1<br>317.2<br>317.3<br>317.4<br>317.5<br>317.7<br>317.8
345	Rural	53.0208371	-3.137519488	1	435.1
356	Rural	52.2343431	0.435041713	9	356.4<br>356.6<br>356.9<br>356.11<br>356.12<br>356.14<br>356.15<br>356.20<br>356.21
366	Rural	51.2635539	-0.264329988	3	366.1-366.3
367	Rural	52.7524236	-3.850037589	1	367.3
409	Rural	50.65161449	-2.007325889	2	409.1 and 409.2
411	Urban	52.5500492	-2.033210288	7	411.1<br>411.2<br>411.3<br>411.4<br>411.5<br>411.6<br>411.7
110a	Urban	51.4452493	-2.598944189	10	110.1<br>110.2<br>110.4<br>110.5<br>110.8<br>110.9<br>110.10<br>110.11<br>110.12 110.14
110b	Urban	51.4452493	-2.598944189	10	110.15<br>110.17<br>110.18<br>110.19<br>110.21<br>110.26<br>110.29<br>110.31<br>110.34<br>110.35

110c	Urban	51.4452493	-2.598944189	10	110.36<br>110.37<br>110.38<br>110.42<br>110.43<br>110.44<br>110.45<br>110.46<br>110.47<br>110.48
110d	Urban	51.4452493	-2.598944189	10	110.49<br>110.50<br>110.52<br>110.53<br>110.55<br>110.56<br>110.57<br>110.58<br>110.59<br>110.60
124a	Urban	53.2879691	-3.210098788	8	124.1<br>124.2<br>124.5<br>124.6<br>124.8<br>124.9<br>124.16<br>124.18
124T	Urban	53.2879691	-3.210098788	1	124.7
131a	Rural	50.87584789	-3.54075249	10	131.1<br>131.2<br>131.4<br>131.5<br>131.6<br>131.8<br>131.9<br>131.10<br>131.11<br>131.14
131b	Rural	50.87584789	-3.54075249	10	131.15<br>131.16<br>131.17<br>131.18<br>131.20<br>131.21<br>131.24<br>131.25<br>131.27<br>131.29
131c	Rural	50.87584789	-3.54075249	10	131.30<br>131.31<br>131.32<br>131.33<br>131.34<br>131.35<br>131.36<br>131.39<br>131.40<br>131.42

131d	Rural	50.87584789	-3.54075249	10	131.44<br>131.46<br>131.47<br>131.48<br>131.50<br>131.53<br>131.56<br>131.58<br>131.62<br>131.67
131e	Rural	50.87584789	-3.54075249	10	131.68<br>131.69<br>131.71<br>131.72<br>131.73<br>131.74<br>131.76<br>131.77<br>131.78<br>131.79
131f	Rural	50.87584789	-3.54075249	10	131.80<br>131.81<br>131.82<br>131.83<br>131.84<br>131.85<br>131.86<br>131.87<br>131.88<br>131.89
132a	Urban	50.72119109	-3.50119829	6	132.1<br>132.2<br>132.3<br><br>132.6<br>132.7<br>132.8
132b	Urban	50.72119109	-3.50119829	7	132.10<br>132.11<br>132.13<br>132.14<br>132.15<br>132.16<br>132.17
132M	Urban	50.72119109	-3.50119829	1	132.5
135a	Rural	53.160586	-2.590867588	8	135.6<br>135.7<br>135.9<br>135.14<br>135.16<br>135.18<br>135.19<br>135.22
135b	Rural	53.160586	-2.590867588	7	135.24<br>135.30<br>135.35<br>135.38<br>135.39<br>135.42<br>135.43

143a	Rural	50.88703829	-2.498572389	10	143.1-10
143b	Rural	50.88703829	-2.498572389	10	143.11-143.16<br>143.18<br>143.19<br>143.21<br>143.23
143c	Rural	50.88703829	-2.498572389	10	143.24-143.34
143d	Rural	50.88703829	-2.498572389	10	143.35<br>143.36<br>143.37<br>143.38<br>143.39<br>143.41<br>143.42<br>143.43<br>143.44<br>143.48
148T	Rural	54.4771787	-1.728842687	2	148.1 and 148.3
155a	Rural	52.6769632	-1.688938888	7	155.3<br>155.4<br>155.6<br>155.8<br>155.9<br>155.10<br>155.11
155b	Rural	52.6769632	-1.688938888	6	155.13<br>155.15<br>155.16<br>155.17<br>155.18<br>155.19
156a	Urban	52.4764236	-1.873005388	8	156.3<br>156.4<br>156.5<br>156.7<br>156.8<br>156.9<br>156.11<br>156.12
156b	Urban	52.4764236	-1.873005388	8	156.13<br>156.14<br>156.15<br>156.16<br>156.18<br>156.19<br>156.20<br>156.21
159a	Rural	51.6881176	-2.094882388	6	159.3 159.4 159.5 159.7
159T	Rural	51.6881176	-2.094882388	4	159.2 159.6 159.8 159.10
160a	Urban	51.5775564	-1.774488988	10	160.1-160.10
160b	Urban	51.5775564	-1.774488988	10	160.11<br>160.12<br>160.13<br>160.15<br>160.16<br>160.17<br>160.18<br>160.19<br>160.20<br>160.21

160c	Urban	51.5775564	-1.774488988	10	160.22<br>160.23<br>160.24<br>160.26<br>160.27<br>160.28<br>160.29<br>160.32<br>160.36<br>160.37
160d	Urban	51.5775564	-1.774488988	10	160.38<br>160.41<br>160.42<br>160.43<br>160.44<br>160.45<br>160.46<br>160.47<br>160.48<br>160.49
161a	Rural	51.405834	-1.833680988	8	161.3<br>161.4<br><br>161.8<br>161.9<br>161.10<br>161.12<br>161.13<br><br>161.16
161T	Rural	51.405834	-1.833680988	2	161.14 and 161.15
166a	Urban	54.5628542	-1.230693187	10	166.1<br>166.2<br>166.3<br>166.4<br>166.5<br>166.6<br>166.7<br>166.8<br>166.9<br>166.11
166b	Urban	54.5628542	-1.230693187	10	166.12<br>166.13<br>166.14<br>166.15<br>166.17<br>166.18<br>166.19<br>166.20<br>166.21<br>166.22
166c	Urban	54.5628542	-1.230693187	10	166.23<br>166.24<br>166.25<br>166.26<br>166.27<br>166.28<br>166.29<br>166.30<br>166.31<br>166.32

166d	Urban	54.5628542	-1.230693187	10	166.33<br>166.34<br>166.35<br>166.36<br>166.38<br>166.39<br>166.40<br>166.41<br>166.42<br>167.1
172a	Urban	52.9600655	-1.189299387	7	172.2<br>172.4<br>172.5<br>172.6<br>172.7<br>172.8<br>172.9
172b	Urban	52.9600655	-1.189299387	6	172.10<br>172.11<br>172.14<br>172.16<br>172.18<br>172.19
174a	Urban	52.6353423	-1.108659287	9	174.2<br>174.3<br>174.4<br>174.5<br>174.7<br>174.8<br>174.9<br>174.10<br>174.11
174b	Urban	52.6353423	-1.108659287	9	174.12<br>174.13<br>174.14<br>174.15<br>174.17<br>174.21<br>174.22<br>174.23<br>174.25
178a	Rural	51.7506107	-1.228417588	9	178.1<br>178.2<br>178.6<br>178.7<br>178.9<br>178.10<br>178.12<br>178.13<br>178.14
186a	Urban	53.747174	-0.371642686	8	186.4<br>186.5<br>186.6<br>186.9<br>186.10<br>186.12<br>186.14<br>186.15

186b	Urban	53.747174	-0.371642686	10	186.16<br>186.17<br>186.18<br>186.19<br>186.20<br>186.21<br>186.22<br>186.23<br>186.24<br>186.25
186d	Urban	53.747174	-0.371642686	10	186.38<br>186.39<br>186.40<br>186.41<br>186.42<br>186.43<br>186.44<br>186.45<br>186.46<br>186.49
193a	Suburban	51.5671309	-0.395082388	7	193.1<br>193.2<br>193.3<br>193.4<br>193.5<br>193.6<br>193.7
193b	Suburban	51.5671309	-0.395082388	7	193.8 193.9 193.10<br>193.12-193.15
204a	Rural	52.2073267	0.133894313	9	204.1<br>204.3<br>204.5<br>204.7<br>204.8<br>204.9<br>204.10<br>204.11<br>204.12
208a	Urban	51.4925508	-0.085819487	9	208.1<br>208.2<br>208.3<br>208.4<br>208.5<br>208.6<br>208.9<br>208.12<br>208.13
208b	Urban	51.4925508	-0.085819487	9	208.15<br>208.16<br>208.17<br>208.21<br>208.22<br>208.23<br>208.24<br>208.25<br>208.26

214a	Rural	52.0411092	1.206661813	9	214.1<br>214.3<br>214.4<br>214.5<br>214.6<br>214.7<br>214.10<br>214.11<br>214.13
214b	Rural	52.0411092	1.206661813	9	214.14<br>214.15<br>214.16<br>214.17<br>214.19<br>214.20<br>214.21<br>214.22<br>214.23
214M	Rural	52.0411092	1.206661813	1	214.18
215a	Rural	51.6783528	0.874142513	10	215.2<br>215.4<br>215.6<br>215.18<br>215.19<br>215.21<br>215.22<br>215.24<br>215.25<br>215.26
215b	Rural	51.6783528	0.874142513	10	215.29<br>215.30<br>215.31<br>215.32<br>215.33<br>215.36<br>215.37<br>215.38<br>215.39<br>215.40
215d	Rural	51.6783528	0.874142513	9	215.60<br>215.64<br>215.65<br>215.66<br>215.70<br>215.73<br>215.74<br>215.75<br>215.76
215e	Rural	51.6783528	0.874142513	9	215.85<br>215.86<br>215.88<br>215.89<br>215.90<br>215.91<br>215.93<br>215.95<br>215.96

216a	Urban	51.5421848	0.702470513	6	216.1<br>216.3<br>216.4<br>216.5<br>216.7<br>216.10
216T	Urban	51.5421848	0.702470513	1	216.9
302a	Rural	53.7446821	-2.936278688	9	302.4 302.6 302.7
302T	Rural	53.7446821	-2.936278688	1	302.3
304a	Rural	52.5229257	-3.992387889	7	304.1<br>304.4<br>304.5<br>304.6<br>304.8<br>304.9<br>304.10
304b	Rural	52.5229257	-3.992387889	8	304.11<br>304.12<br>304.13<br>304.14<br>304.15<br>304.18<br>304.19 304.21
304c	Rural	52.5229257	-3.992387889	8	304.24<br>304.25<br>304.26<br>304.28<br>304.29<br>304.30<br>304.34<br>304.36
319b	Rural	51.7412479	-2.403051589	10	319.119<br>319.120<br>319.121<br>319.122<br>319.123<br>319.124<br>319.125<br>319.127<br>319.128<br>319.129
319c	Rural	51.7412479	-2.403051589	10	319.130<br>319.131<br>319.132<br>319.135<br>319.136<br>319.137<br>319.138<br>319.139<br>319.140<br>319.142
319d	Rural	51.7412479	-2.403051589	10	319.143<br>319.144<br>319.145<br>319.146<br>319.147<br>319.148<br>319.149<br>319.150<br>319.151<br>319.152

320T	Rural	55.177232	-2.461576487	2	320.1 320.2
322a	Rural	51.4456567	0.623335413	8	322.3<br>322.4<br>322.5<br>322.6<br>322.9<br>322.10<br>322.11<br>322.12
322b	Rural	51.4456567	0.623335413	7	322.13<br>322.14<br>322.15<br>322.17<br>322.18<br>322.19<br>322.20
322c	Rural	51.4456567	0.623335413	7	322.21<br>322.22<br>322.24<br>322.25<br>322.26<br>322.27<br>322.28
324a	Rural	51.4391182	0.395436013	10	324.28<br>324.29<br>324.30<br>324.31<br>324.32<br>324.34<br>324.35<br>324.36<br>324.37<br>324.38
324b	Rural	51.4391182	0.395436013	10	324.18<br>324.19<br>324.20<br>324.21<br>324.22<br>324.23<br>324.24<br>324.25<br>324.26<br>324.27
324c	Rural	51.4391182	0.395436013	10	324.7<br>324.8<br>324.9<br>324.1<br>324.11<br>324.12<br>324.13<br>324.14<br>324.16<br>324.17
325a	Rural	50.69504719	-1.930172789	9	325.1<br>325.5<br>325.6<br>325.7<br>325.9<br>325.11<br>325.15<br>325.16<br>325.17

325b	Rural	50.69504719	-1.930172789	9	325.18<br>325.19<br>325.20<br>325.21<br>325.22<br>325.23<br>325.24<br>325.25<br>325.26
325c	Rural	50.69504719	-1.930172789	10	325.26<br>325.28<br>325.30<br>325.31<br>325.33<br>325.36<br>325.37<br>325.38<br>325.39<br>325.41
330T	Rural	53.1672985	-4.064333189	1	330.1 (320.1)
352a	Rural	52.3319465	-3.539158789	1	352.1
352T	Rural	52.3319465	-3.539158789	1	352.2
354b	Rural	51.2314353	-2.186900389	9	354.11<br>354.12<br>354.13<br>354.16<br>354.17<br>354.18<br>354.19<br>354.20<br>354.21
354c	Rural	51.2314353	-2.186900389	8	354.22<br>354.23<br>354.24<br>354.25<br>354.26<br>354.27<br>354.28<br>354.29
354M	Rural	51.2314353	-2.186900389	1	354.9
360a	Rural	53.8504427	-0.931272087	9	360.1<br>360.2<br>360.3<br>360.5<br>360.6<br>360.7<br>360.8<br>360.10<br>360.11<br>360.12
360b	Rural	53.8504427	-0.931272087	9	360.13<br>360.14<br>360.15<br>360.19<br>360.20<br>360.21<br>360.22<br>360.23<br>360.25

364a	Rural	51.1396754	-0.781913888	10	364.1<br>364.2<br>364.3<br>364.5<br>364.7<br>364.8<br>364.9<br>364.10<br>364.11<br>364.12
364T	Rural	51.1396754	-0.781913888	3	364.4 364.6 364.13
365a	Rural	51.2452764	0.104605912	8	365.1<br>365.3<br>365.4<br>365.5<br>365.8<br>365.9<br>365.11<br>365.12
365b	Rural	51.2452764	0.104605912	7	365.13<br>365.14<br>365.18<br>365.20<br>365.21<br>365.22<br>365.23
403a	Suburban	51.4150035	-0.108357588	8	403.2<br>403.3<br>403.4<br>403.5<br>403.6<br>403.7<br>403.8<br>403.9
403b	Suburban	51.4150035	-0.108357588	8	403.10<br>403.11<br>403.12<br>403.13<br>403.14<br>403.15<br>403.16<br>403.17
403c	Suburban	51.4150035	-0.108357588	9	403.18<br>403.20<br>403.21<br>403.24<br>403.25<br>403.26<br>403.27<br>403.28<br>403.29
413a	Rural	51.9615842	0.346123713	7	413.2<br>413.3<br>413.4<br>413.5<br>413.6<br>413.7<br>413.9

413b	Rural	51.9615842	0.346123713	7	413.10<br>413.12<br>413.13<br>413.15<br>413.16<br>413.17<br>413.18
413c	Rural	51.9615842	0.346123713	8	413.19<br>413.20<br>413.21<br>413.22<br>413.23<br>413.24<br>413.25<br>413.28
Neg_control_1	NA	NA	NA	NA	
Pos_control_1	NA	NA	NA	1	

Supplementary Table S2. Pools per site and association tests with geography

SiteID	LandType	Latitude	Longitude	Pools per site
104	Urban	51.615613	-3.94996639	1
105	Rural	53.324464	-4.42686669	1
110	Urban	51.445249	-2.59894419	4
113	Rural	50.546367	-4.31608799	1
114	Urban	50.385989	-4.11885559	1
118	Urban	54.540393	-3.56248059	1
119	Rural	53.885502	-2.73606119	1
120	Urban	54.041129	-2.79150119	1
122	Rural	53.404711	-2.93335759	1
123	Rural	53.225294	-3.48002479	1
124	Urban	53.287969	-3.21009879	2
130	Urban	51.481667	-3.18895669	1
131	Rural	50.875848	-3.54075249	6
132	Urban	50.721191	-3.50119829	3
135	Rural	53.160586	-2.59086759	2
136	Urban	53.254036	-2.51439259	1
138	Urban	52.702516	-2.50851089	1
139	Rural	52.233238	-2.58082979	1
142	Urban	51.589479	-2.97568049	1
143	Rural	50.887038	-2.49857239	4
144	Urban	50.626876	-2.47246479	1
148	Rural	54.477179	-1.72884269	1
149	Rural	53.971692	-2.17599879	1
150	Urban	53.794388	-1.25911329	1
154	Urban	53.053945	-2.19541989	1
155	Rural	52.676963	-1.68893889	2
156	Urban	52.476424	-1.87300539	2
157	Rural	52.29498	-2.20940109	1
159	Rural	51.688118	-2.09488239	2
160	Urban	51.577556	-1.77448899	4
161	Rural	51.405834	-1.83368099	2
162	Urban	51.087065	-1.79466999	1
164	Urban	50.725353	-1.85596579	1
166	Urban	54.562854	-1.23069319	4
167	Rural	54.072108	-1.28442889	1
168	Urban	53.800259	-1.51881309	1
172	Urban	52.960066	-1.18929939	2
173	Rural	52.6788	-1.05354099	1
174	Urban	52.635342	-1.10865929	2
178	Rural	51.750611	-1.22841759	1
179	Rural	51.250363	-1.51212799	1
180	Urban	51.393827	-1.31908749	1
181	Rural	50.857115	-1.59152969	1
182	Urban	50.936763	-1.37651509	1
186	Urban	53.747174	-0.37164269	3
188	Urban	53.208838	-0.55712179	1
190	Urban	52.58343	-0.23732239	1
191	Rural	52.277317	-0.48799329	1
193	Suburban	51.567131	-0.39508239	2
194	Urban	51.542187	-0.24365119	1
195	Suburban	51.387769	-0.23337419	1
196	Urban	51.495037	-0.27084099	1
197	Rural	50.754136	-0.76563509	1
198	Urban	50.840297	-0.76114219	1
204	Rural	52.207327	0.13389431	1
206	Urban	51.572976	-0.09606979	1
208	Urban	51.492551	-0.08581949	2
211	Rural	50.921306	0.33608971	1
212	Urban	52.633003	1.30921381	1
214	Rural	52.041109	1.20666181	3
215	Rural	51.678353	0.87414251	4
216	Urban	51.542185	0.70247051	2

LandType	n sites	median pools	IQR pools	min pools	max pools
Urban	36	1	1–2	1	4
Rural	54	1	1–2	1	6
Suburban	3	2	2–2	1	3
Overall	93	1	1–2	1	6

Test	Comparison	Statistic	df	p value
Kruskal–Wallis	Pools per site ~ LandType (Urban/Rural/Suburban)	H = 1.455	2	0.483
Mann–Whitney U	Pools per site ~ LandType (Urban vs Rural)	U = 906		0.523
Spearman rank	Pools per site vs Latitude	ρ = -0.156		0.136
Spearman rank	Pools per site vs Longitude	ρ = 0.124		0.238

218	Urban	51.378828	1.39465711	1
301	Rural	52.902174	0.00647641	1
302	Rural	53.744682	-2.93627869	2
303	Rural	54.084175	-3.23774819	1
304	Rural	52.522926	-3.99238789	3
305	Rural	53.888457	-3.02996379	1
306	Rural	51.853794	1.22300171	1
307	Rural	50.537738	-4.93477769	1
308	Rural	52.887612	-4.10768999	1
310	Rural	54.750528	-3.44700879	1
314	Rural	55.674265	-1.80067689	1
317	Rural	51.457697	-0.54709509	1
319	Rural	51.741248	-2.40305159	3
320	Rural	55.177232	-2.46157649	1
322	Rural	51.445657	0.62333541	3
324	Rural	51.439118	0.39543601	3
325	Rural	50.695047	-1.93017279	3
330	Rural	53.167299	-4.06433319	1
345	Rural	53.020837	-3.13751949	1
352	Rural	52.331947	-3.53915879	2
354	Rural	51.231435	-2.18690039	3
356	Rural	52.234343	0.43504171	1
360	Rural	53.850443	-0.93127209	2
364	Rural	51.139675	-0.78191389	2
365	Rural	51.245276	0.10460591	2
366	Rural	51.263554	-0.26432999	1
367	Rural	52.752424	-3.85003759	1
403	Suburban	51.415004	-0.10835759	3
409	Rural	50.651614	-2.00732589	1
411	Urban	52.550049	-2.03321029	1
413	Rural	51.961584	0.34612371	3

Supplementary Table S3. Summary of virus presence across sites

Virus_name	n_sites_present	n_ITL_present	sites_present	ITLs_present	Prevalence
Daeseongdong virus 2	70	10	104, 113, 114, 131, 119, 120, 122, 123, 124, 130, 132, 110, 135, 136, 138, 142, 143, 154, 155, 156, 157, 144, 354, 159, 409, 166, 167, 168, 172, 174, 178, 324, 186, 188, 190, 193, 195, 196, 204, 206, 180, 301, 413, 356, 216, 215, 218, 302, 303, 304, 325, 308, 310, 314, 317, 366, 365, 364, 164, 162, 330, 160, 360, 211, 367, 403, 411, 214, 306, 212	TLL, TLK, TLD, TLG, TLC, TLE, TLF, TLJ, TLH, TLI	Common
Wuhan Mosquito virus 4	38	10	105, 131, 124, 130, 132, 110, 138, 142, 143, 149, 156, 319, 166, 172, 174, 178, 322, 324, 186, 188, 193, 204, 206, 180, 413, 215, 302, 303, 325, 314, 365, 364, 164, 161, 160, 360, 211, 214	TLL, TLK, TLG, TLE, TLC, TLF, TLJ, TLI, TLH, TLD	Common
Alphamesonivirus fluvideense	36	10	104, 113, 114, 119, 132, 110, 139, 142, 143, 319, 168, 172, 174, 322, 324, 186, 188, 190, 195, 196, 204, 206, 208, 180, 301, 356, 304, 308, 310, 325, 330, 160, 360, 211, 198, 403	TLL, TLK, TLD, TLG, TLE, TLF, TLJ, TLH, TLI, TLC	Common
Chrysoviridae sp.	25	8	131, 130, 132, 110, 138, 143, 149, 155, 156, 157, 319, 354, 166, 174, 322, 324, 191, 190, 215, 304, 325, 365, 161, 360, 214	TLK, TLL, TLG, TLE, TLC, TLF, TLJ, TLH	Intermediate
Wuhan Mosquito virus 6	19	8	113, 131, 150, 354, 159, 409, 166, 174, 322, 324, 206, 215, 302, 305, 325, 365, 164, 360, 214	TLK, TLE, TLC, TLF, TLJ, TLI, TLH, TLD	Intermediate
Marma virus	18	6	131, 110, 143, 319, 409, 186, 191, 190, 193, 208, 413, 215, 310, 365, 164, 214, 360, 197	TLK, TLE, TLH, TLI, TLD, TLJ	Intermediate
Culex mononega-like virus 1	17	9	131, 136, 354, 159, 166, 168, 172, 322, 324, 186, 196, 304, 325, 365, 164, 360, 306	TLK, TLD, TLC, TLE, TLF, TLJ, TLI, TLL, TLH	Intermediate
Culex Negev-like virus 1	16	8	132, 142, 172, 174, 186, 195, 196, 324, 204, 208, 180, 304, 317, 160, 211, 403	TLK, TLL, TLF, TLE, TLI, TLJ, TLH, TLD	Intermediate
Culex Negev-like virus 2	15	8	132, 142, 172, 174, 186, 195, 196, 324, 204, 208, 180, 317, 160, 211, 403	TLK, TLL, TLF, TLE, TLI, TLJ, TLH, TLD	Intermediate
Culex pipiens Ifla-like virus 2	15	4	132, 110, 143, 319, 354, 409, 204, 208, 215, 218, 364, 211, 403, 160, 214	TLK, TLH, TLI, TLJ	Intermediate
Culex mononega-like virus 2	14	7	132, 139, 143, 148, 149, 354, 159, 215, 325, 320, 364, 161, 345, 160	TLK, TLG, TLE, TLH, TLC, TLJ, TLL	Intermediate
Ghabrivirales sp. 1	13	6	131, 132, 110, 142, 143, 319, 322, 186, 216, 215, 325, 211, 403	TLK, TLL, TLJ, TLE, TLH, TLI	Intermediate
Ista virus	12	6	105, 131, 119, 120, 135, 149, 354, 215, 304, 310, 320, 162	TLL, TLK, TLD, TLE, TLH, TLC	Intermediate
Merida virus	10	7	120, 122, 123, 110, 156, 319, 354, 178, 186, 413	TLD, TLL, TLK, TLG, TLJ, TLE, TLH	Intermediate
Hedwig virus	10	6	104, 110, 142, 319, 174, 322, 194, 208, 356, 214	TLL, TLK, TLF, TLJ, TLI, TLH	Intermediate
Ghabrivirales sp. 2	10	5	131, 132, 143, 319, 186, 216, 215, 325, 211, 403	TLK, TLE, TLH, TLJ, TLI	Intermediate
Jotan virus	8	5	110, 143, 159, 172, 191, 325, 320, 403	TLK, TLF, TLH, TLC, TLI	Intermediate
Jotan-like virus	7	4	110, 143, 159, 172, 325, 320, 403	TLK, TLF, TLC, TLI	Intermediate
Culex circovirus-like virus	6	5	132, 142, 188, 195, 196, 180	TLK, TLL, TLF, TLI, TLJ	Intermediate
Cripavirus pipiens	4	4	156, 178, 208, 215	TLG, TLJ, TLI, TLH	Rare
Culex Negev-like virus 4	4	4	166, 322, 215, 164	TLC, TLJ, TLH, TLK	Rare
Culex Sobemo-like virus	4	3	143, 188, 191, 161	TLK, TLF, TLH	Rare
Culex bunyavirus 2	4	3	131, 167, 413, 215	TLK, TLE, TLH	Rare
Culex mosquito virus 4	4	3	110, 186, 215, 214	TLK, TLE, TLH	Rare
Culex pipiens Tymo-like virus 2	4	3	120, 317, 366, 161	TLD, TLJ, TLK	Rare
Culex pipiens Tymo-like virus 3	3	2	105, 131, 144	TLL, TLK	Rare
Umatilla virus	3	2	114, 142, 319	TLK, TLL	Rare
Atherstone virus	2	2	204, 160	TLH, TLK	Rare
Culex luteo-like virus	2	2	135, 159	TLD, TLK	Rare
Culex pipiens Ifla-like virus 1	2	2	188, 161	TLF, TLK	Rare
Culex pipiens deltapartitivirus	2	2	191, 304	TLH, TLL	Rare
Culex pipiens nodavirus strain	2	2	218, 162	TLJ, TLK	Rare
Tolivirales sp. 2	2	2	188, 197	TLF, TLJ	Rare
Almendravirus Chester	1	1	161	TLK	Rare
Amalgaviridae sp.	1	1	143	TLK	Rare
Culex Negev-like virus 3	1	1	215	TLH	Rare
Culex pipiens Tymo-like virus 1	1	1	155	TLG	Rare
Culex pipiens Tymovirales sp.	1	1	413	TLH	Rare
Culex pipiens betapartitivirus 2	1	1	143	TLK	Rare
Tolivirales sp. 1	1	1	168	TLE	Rare
Valmbacken virus	1	1	110	TLK	Rare

Supplementary Table S4. Site-level relative abundance (% of viral reads) and library occurrence of detected viruses (n = 151 libraries).

Virus name	Number of libraries present (max 151)	Median site-level relative abundance (% of viral reads)	Minimum site-level relative abundance (% of viral reads)	Maximum site-level relative abundance (% of viral reads)
Almendravirus Chester	1	84.52515025	84.52515025	84.52515025
Merida virus	10	84.01446371	1.251139783	99.97591727
Ista virus	15	45.71174201	3.99E-04	100
Culex pipiens Tymo-like virus 1	2	39.88248572	29.43211025	50.33286119
Tolivirales sp. 2	2	35.41974635	0.006159366	70.83333333
Culex Negev-like virus 4	4	32.97320672	0.785169198	93.82287979
Culex pipiens Tymo-like virus 3	3	31.64314248	30.70036601	98.09174872
Umatilla virus	3	29.47931619	0.0081747	60
Culex pipiens Ifla-like virus 2	16	21.84693463	0.036747582	99.29546837
Alphamesonivirus fluvideense	55	21.74916514	0.003608716	100
Culex mosquito virus 4	4	21.64536958	0.00264095	67.82818714
Culex luteo-like virus	2	20.72948889	0.267956824	41.19102096
Jotan-like virus	8	20.0501784	0.014175357	62.8897406
Jotan virus	8	11.44235739	0.859585995	71.38836409
Chrysoviridae sp.	35	9.783995448	0.014890601	99.50140521
Tolivirales sp. 1	1	9.504761323	9.504761323	9.504761323
Culex pipiens deltapartitivirus	2	8.100929312	0.097297798	16.10456083
Marma virus	21	6.922980667	7.69E-04	100
Amalgaviridae sp.	2	5.040022184	0.806808755	9.273235613
Culex Negev-like virus 2	20	4.499538736	0.143936659	32.82625851
Culex Negev-like virus 1	21	4.024205132	0.004891174	41.57508681
Daeseongdong virus 2	96	3.823104823	1.14E-04	100
Cripavirus pipiens	4	3.52591078	0.303411071	6.861238786
Culex Negev-like virus 3	3	2.971551352	0.00753695	100
Culex mononega-like virus 2	15	2.536221905	0.005715963	100
Culex pipiens Tymo-like virus 2	5	2.440775815	0.008125918	94.72025217
Wuhan Mosquito virus 6	23	2.309484876	2.24E-04	100
Culex bunyavirus 2	5	2.16377792	0.659501234	99.50856844
Culex mononega-like virus 1	21	1.673307657	0.001171285	99.96180352
Ghabrivirales sp. 2	10	1.603411741	0.034693672	40.79203636
Culex pipiens betapartitivirus	3	1.040087823	0.151206417	3.74290932
Wuhan Mosquito virus 4	53	0.912318432	2.24E-04	100
Ghabrivirales sp. 1	14	0.527903335	8.19E-04	83.33333333
Valmbacken virus	1	0.346497894	0.346497894	0.346497894
Culex pipiens Ifla-like virus 1	2	0.321586067	0.015391445	0.627780689
Culex pipiens nodavirus	2	0.216353226	0.074478055	0.358228398
Hedwig virus	11	0.212504481	4.14E-04	100
Culex Sobemo-like virus	4	0.114155306	9.76E-05	1.572068367
Culex pipiens Tymovirales sp.	1	0.031580614	0.031580614	0.031580614
Atherstone virus	2	0.005117807	0.004529772	0.005705843
Culex circovirus-like virus	7	0.003253445	6.10E-05	0.102466579

Supplementary Table S5. Number of sites containing viral families

Taxa	Number_sites_detected
Unclassified	73
Orthomyxoviridae	44
Mesoniviridae	36
Iflaviridae	31
Xinmoviridae	26
Chrysoviridae	25
Solemoviridae	22
Peribunyaviridae	12
Rhabdoviridae	11
Tymoviridae	8
Draupnirviridae	6
Sedoreoviridae	4
Dicistoviridae	4
Chuviridae	4
Partitiviridae	3
Nodaviridae	2
Amalgaviridae	1

Supplementary Table S6. Viral family composition across rural, urban, and suburban sites, with DESeq2 filtering status and differential abundance statistics (Rural vs Urban).

Virus_Family	Rural	Urban	Suburban	Total	Pass filter for Deseq2 analysis	Mean_normalised_counts	Log2_fold_change_(Rural_vs_Urban)	SE_of_log2_fold_change	Wald_statistic	Raw_p_value	Adjusted_p_value_(FDR)	Enriched_in_condition
Amalgaviridae	1	0	0	1	FALSE	NA	NA	NA	NA	NA	NA	NA
Chrysoviridae	16	9	0	25	TRUE	1129.751326	-1.837605712	1.762220455	-1.042778562	0.297050862	0.415871207	NA
Chuviridae	1	3	0	4	FALSE	NA	NA	NA	NA	NA	NA	NA
Dicistoviridae	1	3	0	4	FALSE	NA	NA	NA	NA	NA	NA	NA
Draupnirviridae	0	5	1	5	FALSE	NA	NA	NA	NA	NA	NA	NA
Iflaviridae	19	11	1	30	TRUE	36786.01814	1.135707924	1.637534878	0.693547319	0.487966141	0.525501998	NA
Mesoniviridae	16	18	2	34	TRUE	706280.5507	5.8070286	1.53421145	3.785024939	1.54E-04	5.38E-04	Urban
Nodaviridae	0	2	0	2	FALSE	NA	NA	NA	NA	NA	NA	NA
Orthomyxoviridae	23	20	1	43	TRUE	7010.887053	-1.714388638	1.210494492	-1.416271324	0.15669608	0.365624187	NA
Partitiviridae	3	0	0	3	FALSE	NA	NA	NA	NA	NA	NA	NA
Peribunyaviridae	3	9	0	12	FALSE	NA	NA	NA	NA	NA	NA	NA
Rhabdoviridae	6	5	0	11	FALSE	NA	NA	NA	NA	NA	NA	NA
Sedoreoviridae	1	3	0	4	FALSE	NA	NA	NA	NA	NA	NA	NA
Solemoviridae	14	7	1	21	FALSE	NA	NA	NA	NA	NA	NA	NA
Tymoviridae	6	2	0	8	FALSE	NA	NA	NA	NA	NA	NA	NA
Unclassified	37	34	3	71	TRUE	630409.4362	0.93505463	0.838666382	1.114930382	0.264880256	0.413204167	NA
Xinmoviridae	19	9	0	28	TRUE	3431.850601	-4.119162171	1.621951108	-2.539633994	0.011096853	0.031071188	Rural

Supplementary Table S7. Virus occurrence across rural, suburban, and urban sites, with DESeq2 filtering status and differential abundance results (Rural vs Urban).

Virus_name	Rural	Suburban	Urban	Total	Pass_filter	Mean_normalised_counts	Log2_fold_change_(Rural_vs_Urban)	SE_of_log2_fold_change	Wald_statistic	Raw_p_value	Adjusted_p_value_(FDR)	Enriched_in_condition
Almendravirus Chester	1	0	0	1	FALSE	NA	NA	NA	NA	NA	NA	NA
Alphamesonivirus fluvideense	16	2	18	34	TRUE	276846.8899	3.657826915	1.562562544	2.340915523	0.019236519	0.051297384	NA
Amalgaviridae sp.	1	0	0	1	FALSE	NA	NA	NA	NA	NA	NA	NA
Atherstone virus	0	0	2	2	FALSE	NA	NA	NA	NA	NA	NA	NA
Chrysoviridae sp.	16	0	9	25	TRUE	2617.159592	-1.41285534	1.859197981	-0.75992732	0.44729803	0.536757636	NA
Cripavirus pipiens	1	0	3	4	FALSE	NA	NA	NA	NA	NA	NA	NA
Culex bunyavirus 2	4	0	0	4	FALSE	NA	NA	NA	NA	NA	NA	NA
Culex luteo-like virus	2	0	0	2	FALSE	NA	NA	NA	NA	NA	NA	NA
Culex mononega-like virus 1	10	0	7	17	FALSE	NA	NA	NA	NA	NA	NA	NA
Culex mononega-like virus 2	12	0	2	14	FALSE	NA	NA	NA	NA	NA	NA	NA
Culex mosquito virus 4	1	0	3	4	FALSE	NA	NA	NA	NA	NA	NA	NA
Culex Negev-like virus 1	4	2	10	14	FALSE	NA	NA	NA	NA	NA	NA	NA
Culex Negev-like virus 2	3	2	10	13	FALSE	NA	NA	NA	NA	NA	NA	NA
Culex Negev-like virus 3	1	0	0	1	FALSE	NA	NA	NA	NA	NA	NA	NA
Culex Negev-like virus 4	2	0	2	4	FALSE	NA	NA	NA	NA	NA	NA	NA
Culex pipiens betapartitivirus	1	0	0	1	FALSE	NA	NA	NA	NA	NA	NA	NA
Culex pipiens deltapartitivirus	2	0	0	2	FALSE	NA	NA	NA	NA	NA	NA	NA
Culex pipiens Ifla-like virus 1	1	0	1	2	FALSE	NA	NA	NA	NA	NA	NA	NA
Culex pipiens Ifla-like virus 2	7	1	7	14	FALSE	NA	NA	NA	NA	NA	NA	NA
Culex pipiens nodavirus strain	0	0	2	2	FALSE	NA	NA	NA	NA	NA	NA	NA
Culex pipiens Tymo-like virus 1	1	0	0	1	FALSE	NA	NA	NA	NA	NA	NA	NA
Culex pipiens Tymo-like virus 2	3	0	1	4	FALSE	NA	NA	NA	NA	NA	NA	NA
Culex pipiens Tymovirales sp. 3	1	0	0	1	FALSE	NA	NA	NA	NA	NA	NA	NA
Culex Sobemo-like virus	3	0	1	4	FALSE	NA	NA	NA	NA	NA	NA	NA
Daeseongdong virus 2	34	3	33	67	TRUE	285412.3102	1.271799169	0.93765378	1.356363293	0.174983606	0.290967505	NA
Ghabrivirales sp. 1	7	1	5	12	FALSE	NA	NA	NA	NA	NA	NA	NA
Ghabrivirales sp. 2	6	1	3	9	FALSE	NA	NA	NA	NA	NA	NA	NA
Hedwig virus	3	0	7	10	FALSE	NA	NA	NA	NA	NA	NA	NA
Ista virus	10	0	2	12	FALSE	NA	NA	NA	NA	NA	NA	NA
Jotan virus	5	1	2	7	FALSE	NA	NA	NA	NA	NA	NA	NA
Jotan-like virus	4	1	2	6	FALSE	NA	NA	NA	NA	NA	NA	NA
Krikovirus	0	1	5	5	FALSE	NA	NA	NA	NA	NA	NA	NA
Marma virus	11	1	6	17	FALSE	NA	NA	NA	NA	NA	NA	NA
Merida virus	5	0	5	10	FALSE	NA	NA	NA	NA	NA	NA	NA
Tolivirales sp. 1	0	0	1	1	FALSE	NA	NA	NA	NA	NA	NA	NA
Tolivirales sp. 2	1	0	1	2	FALSE	NA	NA	NA	NA	NA	NA	NA
Tarnsjo virus	2	0	1	3	FALSE	NA	NA	NA	NA	NA	NA	NA
Umatilla virus	1	0	2	3	FALSE	NA	NA	NA	NA	NA	NA	NA
Valmbacken virus	0	0	1	1	FALSE	NA	NA	NA	NA	NA	NA	NA
Wuhan Mosquito virus 4	18	1	19	37	TRUE	4909.995428	-0.064672022	1.414359068	-0.045725321	0.963529182	0.963529182	NA
Wuhan Mosquito virus 6	13	0	6	19	FALSE	NA	NA	NA	NA	NA	NA	NA

Supplementary Table S8. Fisher's exact test enrichment analysis of virus species occurrence across site categories

Virus_name	p-value	odds_ratio	conf_low	conf_high	p_adj	Enriched_in
Almendravirus Chester	1	0	0	47.32286547	1	NA
Alphamesonivirus fluvideense	0.136136	1.973652396	0.785157308	5.056501128	0.697699	NA
Amalgaviridae sp.	1	0	0	47.32286547	1	NA
Atherstone virus	0.201262	Inf	0.229347023	Inf	0.70641	NA
Chrysoviridae sp.	0.350093	0.599893648	0.203431973	1.679359711	0.897114	NA
Cripavirus pipiens	0.324451	3.793753843	0.291708752	205.6294102	0.886832	NA
Culex bunyavirus 2	0.123926	0	0	1.801899897	0.697699	NA
Culex luteo-like virus	0.499299	0	0	6.456760936	1	NA
Culex mononega-like virus 1	0.791818	0.82174162	0.238339365	2.687557144	1	NA
Culex mononega-like virus 2	0.017703	0.165289775	0.016896325	0.815312906	0.18146	NA
Culex mosquito virus 4	0.324451	3.793753843	0.291708752	205.6294102	0.886832	NA
Culex Negev-like virus 1	0.012136	4.621367568	1.251721658	21.5123104	0.165859	NA
Culex Negev-like virus 2	0.004136	6.274247245	1.523406884	37.47169545	0.141081	NA
Culex Negev-like virus 3	1	0	0	47.32286547	1	NA
Culex Negev-like virus 4	1	1.22230769	0.085116825	17.55502643	1	NA
Culex pipiens betapartitivirus	1	0	0	47.32286547	1	NA
Culex pipiens deltapartitivirus	0.499299	0	0	6.456760936	1	NA
Culex pipiens Ifla-like virus 1	1	1.216891755	0.015177482	97.54267037	1	NA
Culex pipiens Ifla-like virus 2	0.575781	1.47268911	0.419800954	5.29333569	1	NA
Culex pipiens nodavirus strain	0.201262	Inf	0.229347023	Inf	0.70641	NA
Culex pipiens Tymo-like virus 1	1	0	0	47.32286547	1	NA
Culex pipiens Tymo-like virus 2	0.624016	0.393866357	0.00726487	5.121449724	1	NA
Culex pipiens Tymovirales sp.	1	0	0	47.32286547	1	NA
Culex Sobemo-like virus	0.624016	0.393866357	0.00726487	5.121449724	1	NA
Daeseongdong virus 2	0.052279	2.965565037	0.970817455	10.31368594	0.402734	NA
Ghabrivirales sp. 1	1	1.047122096	0.265013427	4.007535004	1	NA
Ghabrivirales sp. 2	1	0.79146203	0.15259573	3.623549084	1	NA
Hedwig virus	0.176415	3.160456057	0.663809213	20.26407187	0.70641	NA
Ista virus	0.058937	0.208044774	0.02091071	1.065777534	0.402734	NA
Jotan virus	0.725345	0.710279545	0.103728419	3.919858225	1	NA
Jotan-like virus	1	0.904846428	0.124954896	5.70218112	1	NA

Culex circovirus-like virus	0.006882	Inf	1.540587178	Inf	0.141081	NA
Marma virus	0.606646	0.729745339	0.214804804	2.327379093	1	NA
Merida virus	0.750502	1.24030995	0.263734833	5.835002762	1	NA
Tolivirales sp. 1	0.451613	Inf	0.031135237	Inf	1	NA
Tolivirales sp. 2	1	1.216891755	0.015177482	97.54267037	1	NA
Culex pipiens Tymo-like virus 3	1	0.600745559	0.009896935	11.92675926	1	NA
Umatilla virus	0.587334	2.475892105	0.124706543	150.2491633	1	NA
Valmbacken virus	0.451613	Inf	0.031135237	Inf	1	NA
Wuhan Mosquito virus 4	0.290304	1.657444986	0.666738389	4.172083967	0.886832	NA
Wuhan Mosquito virus 6	0.206754	0.490867992	0.137488098	1.567840142	0.70641	NA

Supplementary Table S9. Fisher's exact test results for viral family occurrence by site category

Virus Family	p-value	odds_ratio	conf_low	conf_high	p_adj	Enriched_in
Amalgaviridae	1	0	0	47.32287	1	NA
Chrysoviridae	0.350093	0.599894	0.203432	1.67936	0.495965	NA
Chuviridae	0.324451	3.793754	0.291709	205.6294	0.495965	NA
Dicistoviridae	0.324451	3.793754	0.291709	205.6294	0.495965	NA
Draupnirviridae	0.006882	Inf	1.540587	Inf	0.116994	NA
Iflaviridae	0.507693	0.676554	0.252667	1.760833	0.616484	NA
Mesoniviridae	0.136136	1.973652	0.785157	5.056501	0.462864	NA
Nodaviridae	0.201262	Inf	0.229347	Inf	0.495965	NA
Orthomyxoviridae	0.680253	1.214806	0.49616	2.98772	0.770953	NA
Partitiviridae	0.248948	0	0	2.912758	0.495965	NA
Peribunyaviridae	0.032527	4.295754	0.976794	26.51171	0.276482	NA
Rhabdoviridae	1	1.01337	0.22536	4.34518	1	NA
Sedoreoviridae	0.324451	3.793754	0.291709	205.6294	0.495965	NA
Solemoviridae	0.462996	0.625004	0.200378	1.834758	0.605457	NA
Tymoviridae	0.2866	0.378631	0.035457	2.273926	0.495965	NA
Unclassified	0.075314	2.770313	0.834361	10.85626	0.426777	NA
Xinmoviridae	0.115797	0.463169	0.159277	1.266717	0.462864	NA

Supplementary Table S10. Within-species comparisons of alpha diversity (Observed richness and Shannon index) between rural and urban sites using Wilcoxon rank-sum tests, with FDR-adjusted p-values.

Species	Metric	Group1	Group2	Sample_size_group1	Sample_size_group2	Test_statistic	Raw_p_value	Adjusted_p_value_(FDR)	Significance
Culex pipiens	Observed	Rural	Urban	40	36	709	0.913	0.913	ns
Culex pipiens	Shannon	Rural	Urban	40	36	722.5	0.983	0.983	ns
Culex molestus	Observed	Rural	Urban	1	2	1	1	1	ns
Culex molestus	Shannon	Rural	Urban	1	2	1	1	1	ns
Culex torrentium	Observed	Rural	Urban	7	1	3	1	1	ns
Culex torrentium	Shannon	Rural	Urban	7	1	3	1	1	ns

Metric	Group1	Group2	Sample_size_group1	Sample_size_group2	Test_statistic	Raw_p_value	Adjusted_p_value_(FDR)	Significance
Observed	Culex pipiens	Culex molestus	76	3	169	0.161	0.242	ns
Observed	Culex pipiens	Culex torrentium	76	8	437	0.043	0.129	ns
Observed	Culex molestus	Culex torrentium	3	8	10	0.755	0.755	ns
Shannon	Culex pipiens	Culex molestus	76	3	166	0.186	0.297	ns
Shannon	Culex pipiens	Culex torrentium	76	8	389	0.198	0.297	ns
Shannon	Culex molestus	Culex torrentium	3	8	10	0.757	0.757	ns

Supplementary Table S11. PERMDISP (Bray–Curtis) test of homogeneity of multivariate dispersion in viral community composition between land types.

RowID	Distance	UnitID	Species	LandType
104 | C. pipiens	0.531883	104	C. pipiens	Urban
105 | C. pipiens	0.664495	105	C. pipiens	Rural
110 | C. pipiens	0.569316	110	C. pipiens	Urban
113 | C. pipiens	0.620448	113	C. pipiens	Rural
114 | C. pipiens	0.607783	114	C. pipiens	Urban
119 | C. pipiens	0.53774	119	C. pipiens	Rural
120 | C. pipiens	0.739173	120	C. pipiens	Urban
122 | C. pipiens	0.732811	122	C. pipiens	Rural
123 | C. pipiens	0.567318	123	C. pipiens	Rural
124 | C. pipiens	0.710136	124	C. pipiens	Urban
130 | C. pipiens	0.711177	130	C. pipiens	Urban
131 | C. pipiens	0.5272	131	C. pipiens	Rural
132 | C. pipiens	0.681919	132	C. pipiens	Urban
135 | C. pipiens	0.653969	135	C. pipiens	Rural
136 | C. pipiens	0.750754	136	C. pipiens	Urban
138 | C. pipiens	0.69156	138	C. pipiens	Urban
139 | C. pipiens	0.679035	139	C. pipiens	Rural
142 | C. pipiens	0.45595	142	C. pipiens	Urban
143 | C. pipiens	0.705425	143	C. pipiens	Rural
144 | C. pipiens	0.756057	144	C. pipiens	Urban
149 | C. pipiens	0.649	149	C. pipiens	Rural
150 | C. pipiens	0.753854	150	C. pipiens	Urban
154 | C. pipiens	0.572257	154	C. pipiens	Urban
155 | C. pipiens	0.702894	155	C. pipiens	Rural
156 | C. pipiens	0.640311	156	C. pipiens	Urban
157 | C. pipiens	0.703425	157	C. pipiens	Rural
159 | C. pipiens	0.686506	159	C. pipiens	Rural
160 | C. pipiens	0.536909	160	C. pipiens	Urban
161 | C. pipiens	0.671528	161	C. pipiens	Rural
162 | C. pipiens	0.572293	162	C. pipiens	Urban
164 | C. pipiens	0.750215	164	C. pipiens	Urban
166 | C. pipiens	0.746395	166	C. pipiens	Urban
167 | C. pipiens	0.751218	167	C. pipiens	Rural
168 | C. pipiens	0.575577	168	C. pipiens	Urban
172 | C. pipiens	0.43965	172	C. pipiens	Urban
174 | C. pipiens	0.475125	174	C. pipiens	Urban
178 | C. pipiens	0.564681	178	C. pipiens	Urban
180 | C. pipiens	0.477416	180	C. pipiens	Urban
186 | C. pipiens	0.543947	186	C. pipiens	Urban
188 | C. pipiens	0.582634	188	C. pipiens	Urban
190 | C. pipiens	0.690448	190	C. pipiens	Urban
191 | C. pipiens	0.705005	191	C. pipiens	Rural
194 | C. pipiens	0.770023	194	C. pipiens	Urban
196 | C. pipiens	0.556803	196	C. pipiens	Urban
197 | C. pipiens	0.725137	197	C. pipiens	Rural
198 | C. pipiens	0.582794	198	C. pipiens	Urban
204 | C. pipiens	0.700095	204	C. pipiens	Urban
206 | C. pipiens	0.570885	206	C. pipiens	Urban
208 | C. pipiens	0.632819	208	C. pipiens	Urban
211 | C. pipiens	0.714968	211	C. pipiens	Rural
212 | C. pipiens	0.572257	212	C. pipiens	Urban
214 | C. pipiens	0.750282	214	C. pipiens	Urban
215 | C. pipiens	0.710672	215	C. pipiens	Rural
216 | C. pipiens	0.769301	216	C. pipiens	Urban
218 | C. pipiens	0.720044	218	C. pipiens	Urban
301 | C. pipiens	0.594836	301	C. pipiens	Rural
302 | C. pipiens	0.660296	302	C. pipiens	Rural
303 | C. pipiens	0.557226	303	C. pipiens	Rural
304 | C. pipiens	0.664313	304	C. pipiens	Rural
305 | C. pipiens	0.69899	305	C. pipiens	Rural
306 | C. pipiens	0.742876	306	C. pipiens	Rural

LandType	n	mean	sd	se	F_stat	perm_p
Rural	43	0.650876	0.06891	0.010509	1.17662	0.2813
Urban	37	0.630405	0.099029	0.01628	1.17662	0.2813

308|C. pipiens	0.625614	308	C. pipiens	Rural
310|C. pipiens	0.678403	310	C. pipiens	Rural
314|C. pipiens	0.701282	314	C. pipiens	Rural
317|C. pipiens	0.562547	317	C. pipiens	Rural
319|C. pipiens	0.683427	319	C. pipiens	Rural
322|C. pipiens	0.675384	322	C. pipiens	Rural
324|C. pipiens	0.674596	324	C. pipiens	Rural
325|C. pipiens	0.561075	325	C. pipiens	Rural
345|C. pipiens	0.740276	345	C. pipiens	Rural
354|C. pipiens	0.546228	354	C. pipiens	Rural
356|C. pipiens	0.710738	356	C. pipiens	Rural
360|C. pipiens	0.539441	360	C. pipiens	Rural
364|C. pipiens	0.555202	364	C. pipiens	Rural
365|C. pipiens	0.562309	365	C. pipiens	Rural
366|C. pipiens	0.720825	366	C. pipiens	Rural
367|C. pipiens	0.563649	367	C. pipiens	Rural
409|C. pipiens	0.696995	409	C. pipiens	Rural
411|C. pipiens	0.572257	411	C. pipiens	Urban
413|C. pipiens	0.562341	413	C. pipiens	Rural
